# Recent Update of COVID-19 Vaccines

**DOI:** 10.34172/apb.2022.045

**Published:** 2021-10-04

**Authors:** Sameer A. Jadaan, Abdul Waheed Khan

**Affiliations:** ^1^College of Health & Medical Technology, Middle Technical University, Baghdad-Iraq.; ^2^Department of Diabetes, Central Clinical School, Monash University, Victoria-Australia.

**Keywords:** SARS-CoV-2, COVID-19, Coronavirus, COVID-19 vaccines, Clinical trials

## Abstract

Severe acute respiratory syndrome coronavirus type 2 (SARS-CoV-2) has been recently identified as a novel member of beta coronaviruses (CoVs) and the cause of coronavirus disease 2019 (COVID-19). It has been first discovered in China and soon has spread across continents with an escalating number of mortalities. There is an urgent need for developing a COVID-19 vaccine to control the rapid transmission and the deleterious impact of the virus. The potent vaccine should have a good tolerable and efficacious profile to induce target-specific humoral and cellular immune responses. It should also exhibit no or minimal detrimental effects in children, young adults, and elderly people with or without co-morbidities from different racial backgrounds. Previously published findings of SARS-CoV and Middle East respiratory syndrome coronavirus (MERS-CoV) played vital role in the characterization of surface spike proteins as the tool of entry of the SARS-CoV-2 into host cells. It has become evident that SARS-CoVs have high genetic similarity and this implies antecedent vaccination strategies could be implicated in the production of COVID-19 vaccines. Although several vaccines have been approved and rolled out, only a handful of them have passed the three phases of clinical studies. This review highlights the completed, and ongoing clinical trials of COVID-19 vaccines and efforts are being made globally to avert the pandemic.

## Introduction

Coronaviruses (CoVs) are relatively large, enveloped, positive-sense single-stranded RNA viruses, with the largest known genome (ranges from 26–32 kb) amongst all RNA viruses.^1^ CoV surface glycoproteins protrude from the viral membrane in spike-like shapes giving the virus a crown-like appearance, hence the virus is named as Corona. The genome of a CoV is comprised of genes encoding for 16 nonstructural proteins and 4 structural proteins encoded by envelope, membrane, nucleocapsid, and spike genes. Spike (S) protein is significantly important as it is used for the host cell attachment and penetration.^2^ There are seven members of the family Coronaviridae identified in humans. Several CoVs are zoonotic and they are infectious in birds, livestock and other mammals. CoVs can be transmittable crossing species to humans from their natural habitat, possibly through intermediate hosts.^3^ CoVs were previously not considered as highly pathogenic and they mostly caused mild respiratory diseases in human populations. Three CoVs emerged to pose serious threats to public health worldwide. At the beginning of this century, severe acute respiratory syndrome coronavirus (SARS-CoV) was first arisen from the wet market in China and then intensively spread, resulting in a global outbreak.^4^ SARS-CoV is highly infectious to humans with 8098 positive cases and 774 deaths reported.^5^ A decade later, another member Middle East respiratory syndrome coronavirus (MERS-CoV) was discovered in Saudi Arabia from an animal source, camels. It was associated with a higher case fatality rate than SARS-CoV.^6^ At the end of 2019, the world has witnessed the first case of the previously named novel CoV (2019-nCoV) which was later officially designated as COVID-19 in the seafood market in Wuhan, China. It was caused by a newly identified member SARS-CoV-2.^7^ Since its emergence, COVID-19 has become a worldwide major medical threat with current figures of World Health Organization (WHO) showing 115 094 614 confirmed cases, including 2 560 995 deaths.^8^

SARS-CoV-2 is very contagious. Human-to-human transmission occurs via direct physical contact and the inhalation of virally-infected respiratory droplets and aerosols.^9,10^ The mechanism of entry is established when the virus binds to the host receptor angiotensin-converting enzyme 2 (ACE2) to gain entry to the cell after initial priming of the viral S glycoprotein by the trans-membrane protease serine 2 (TMPRSS2). SARS-COV-2 uses the S protein for attachment and penetration into the infected cell.^11^ Once inside the cell, SARS-CoV-2 establishes itself by replicating its genomic RNA, giving rise to new viral copies.

Age and co-morbidities like heart disease, diabetes, pulmonary and kidney diseases are independent risk factors for COVID-19.^12^ SARS-CoV-2 is associated with a variety of clinical presentations. It can be asymptomatic or mild to moderate in many patients, and in other afflicted persons is ranging from common cold-like symptoms, including headache, fever, cough, myalgia, and fatigue to shortness of breath and in some instances gastrointestinal illness.^13,14^ If progressed, COVID-19 could lead to more serious pathologies such as pneumonia, acute respiratory distress, kidney injury, multi-organ dysfunction and may cause death.^15,16^ Infected individuals could encounter an increased incidence of cardiovascular disease due to dysregulated immune responses and excessive inflammation. Disorder in immune system responses during COVID-19 progression and the imbalanced T-helper 1 (Th1) and Th2 dependent signals provoke cytokine storm and the latter may promote myocardial injury.^17,18^

The impact of this pandemic on the livelihood and health of people is inevitable. With no approved treatment, community-based prevention and control strategies have been relatively successful to impede transmission and infection in societies. General hygiene, social distancing, quarantine, and lockdown have been implemented. These measures have been indispensable until the vaccination strategies are applied effectively. As prophylactic immunization is the most efficient way to protect from infections and pandemics, this review article discusses vaccine development and the current knowledge in vaccination strategies and platforms against COVID-19.

## Vaccines development for COVID-19

The clinical trials database (clinicaltrials.gov) was searched using the keywords COVID-19, or SARS-CoV-2 with the default settings. Suspended, terminated, and withdrawn clinical studies were excluded. By the time we drafted this manuscript, 3662 registered clinical studies dedicated for the COVID-19 pandemic were listed on the clinicaltrials.gov website, which have been either completed or are being conducted in 121 countries. Of these, only 2% are vaccine studies at different phases of clinical trials, excluding those listed on WHO International Clinical Trials Registry Platform (ICTP).

While various efforts are being made worldwide to combat the outbreak through safety and precaution measures, we are at a great risk of contracting SARS-CoV-2 infection and the serious consequences associated with it. As a result, the need for an effective vaccine to be deployed on a global scale is crucial.

**Figure 1:**
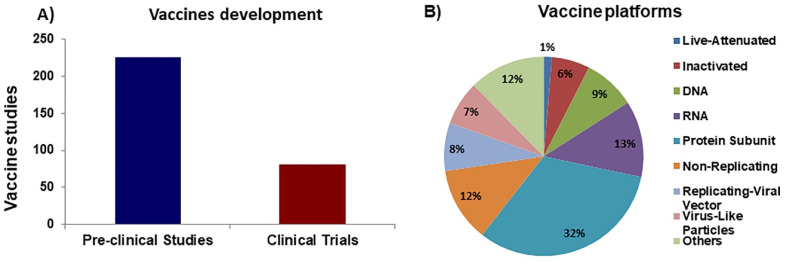
Overview of COVID-19 vaccine development. A) Total number of COVID-19 vaccines currently in development. B) Percentage of platforms (categories) used in vaccine development studies.

The race for COVID-19 vaccines is fierce, but we still far from the finishing line as vaccination of the world population is a very long process. As of November 2020, the COVID-19 vaccine landscape involved (308) candidates with (81) in the clinical evaluation and (227) in the pre-clinical stages (Figure 1A). This review will focus on vaccine candidates that have already entered the clinical phases.

## Strategies of COVID-19 vaccines

Several vaccine-developing strategies have been employed in anti-SARS-CoV-2 vaccine development (Figure 1B), but in general, most of these vaccines are based on 3 main groups of vaccines: whole-pathogen, recombinant subunit, and nucleic acid vaccines. Each of these groups of vaccines has its advantages and drawbacks, which will be summarized. We will also discuss promising candidates from each of these platforms.

### Whole-pathogen vaccines

Those vaccines contain the whole pathogenic particles, whether it is virus or bacterium, and they may be live-attenuated or inactivated (killed) immunogens.

#### Live-attenuated vaccines

Live vaccines are among the most important medical interventions to control disease spread and confer protection against infectious diseases caused by viruses and bacteria. Historically, live vaccines are widely used against viral infections such as poliomyelitis, mumps, rubella, and influenza. They are composed of a weakened (but not killed) version of the pathogenic strains lacking their virulence and pathogenicity arsenal.^19^ They are therefore capable of mimicking the natural infection and stimulating “tailor-fashioned” humoral and cellular immune responses that provide long-lasting protection of the host.^20^ One of the advantages of this kind of vaccine is the robust immunogenicity, which means the immunization can be achieved in 1 or 2 doses. However, they may not be completely safe for immunocompromised individuals as viral replication is probable and clinical manifestations related to infection could occur.^21^ Live-attenuated vaccines targeting COVID-19 are dependent on trained innate immunity and are mostly repurposed immunogens that are originally manufactured to protect against other infections. A list of anti-SARS-CoV-2 live vaccines under investigation from around the globe is discussed in section (Others).

#### Inactivated vaccines

This strategy of vaccine development and manufacture is well-established and there are several prophylactic inactivated vaccines licensed for immunization against viruses. Unlike live-attenuated, inactivated vaccines are produced using a killed version of the infectious agent we need to initiate protection against. For this reason, they are safer than their live-attenuated counterparts.^22^ Generally, vaccines are manufactured by cultivating the pathogen in substrates such as cells, fertilized eggs, or living organisms for virus propagation.^23^ To inactivate the virus, a variety of approaches can be used, involving physical or chemical procedures or both. UV treatment, gamma irradiation, heat, ascorbic acid, and hydrogen peroxide are examples of substances and methods utilized to inactivate viruses.^24,25^ β-Propiolactone and formaldehyde, however, are the most common agents used in the production of inactivated viral vaccines licensed for the use in humans.^26^ In this section, we summarized the most potential COVID-19 inactivated vaccines and elucidated the progress in their development.

## Sinopharm vaccines

The first SARS-CoV-2 chemically inactivated vaccine is co-developed by the Wuhan Institute of Biological Products and Sinopharm. It is based on the WIV04 strain of the virus, which was isolated from a patient in Wuhan, China. It was grown and propagated in Vero cells. The virally infected cells were inactivated using β-propiolactone and the vaccine was adsorbed into alum adjuvant BIBP.

The vaccine is well-tolerated as indicated in the safety analysis of phase I and phase II clinical trials. The adverse effects were mild and transient. Regarding immunogenicity outcomes, in phase II clinical trial, enrolled individuals were randomized to receive 5 μg of the vaccine based on 2 dose regimens. The first group was vaccinated at days 0 and 14, and the second group was injected at days 0 and day 21. In the second group, the mean titer of neutralizing antibodies was almost 2-fold greater than that of the group that received vaccine shots at days 0 and 14. Whereas, IgG binding antibody titers in participants injected at days 0 and 21 were approximately 3-fold higher than titers detected in the group vaccinated at days 0 and 14.^27^ Conclusively, based on these data, the inactivated vaccine exhibited tolerability and elicited robust humoral immunity, but it is unknown whether the vaccine can confer long-term immunity. Phase III clinical studies revealed that two doses of the vaccine have more than 72% efficacy.

The other vaccine is BBIBP-CorV, which is developed by the Beijing Institute of Biological Products in collaboration with Sinopharm based on inactivated virus platform. HB02 strain of SARS-CoV-2 was used to produce the vaccine BBIBP-CorV because of its high replication, genetic stability, and broad-spectrum protection against different strains of the virus. Similar to previously described inactivated vaccines, the purified HB02 was passaged in Vero cells and deactivated by β-propionolactone. A novel production strategy termed “Carrier in a Basket Reactor” was employed for efficient vaccine manufacture. The vaccine is characterized by good immunogenicity as revealed by antibody responses of the immunized mice. Likewise, results were replicable in various tested species involving rats, rabbits, and guinea pigs with 100% seroconversion detected 3 weeks post-vaccination.^28^

A more recent report furtheridentified the safety and immunogenicity characteristics of the vaccine in extended cohorts who participated in phase I/II trials with 100% of recipients showed virus-specific humoral responses. At 4 µg/dose, BBIBP-CorV was highly immunogenic in adults who received the boost vaccination 3 weeks from the first injection.^29^ However, it is critical to know whether the investigational vaccine can elicit T cell-mediated responses and if the protective effect can be sustained for a long period. It is worthwhile to mention that this vaccine is the first to be approved in China and is now approved in many other countries.

## COVAXIN (BBV152)

Bharat Biotech and Indian Council of Medical Research developed a whole virion inactivated COVID-19 vaccine, which was derived from an isolated SARS-COV-2 strain NIV-2020-770, propagatedin the CCL-81 Vero cells, and inactivated by β-propiolactone.

Safety and immunogenicity testing was conducted on three species; mice, rats, and rabbits. Two different vaccine formulations were applied, a 6 μg with the adjuvant aluminium hydroxide gel (Algel), and a 3 μg and 6 μg adjuvanted with a TLR7/8 agonist adsorbed on Algel.The study found that BBV152 vaccine concentrations in both formulations were well-tolerated and elicited potent antibody activity in all three species.The vaccine formulation with the adjuvant TLR7/8 agonist produced elevated levels of virus-specific interferon gamma (IFN-γ) secreting CD4+ T lymphocytes, suggestive for Th1-skewed responses and cellular mediated protective immunity.^30^ In agreement with these findings, BBV152 protected macaques exposed to SARS-CoV-2 via enhancing specific humoral immune responses. In addition, it successfully enhanced viral clearance a week after natural infection with no pulmonary pathology detected in tested animal tissues.^31^ Clinical studies have also shown encouraging results. Phase I clinical trial demonstrated that COVAXIN® has good safety and immunogenicity profiles. Phase III multi-Center clinical trials have started and the efficacy of the vaccine is being assessed. In January 2021, COVAXIN® has been given approval for the restricted emergency use in India. The vaccine is in a liquid form and therefore it requires no reconstitution, and it can be stored at 2-8°C.

## PiCoVacc (Sinovac Biotech)

The vaccine (also named Coronavac) is engineered using a traditional method of vaccine development, the chemical inactivation of the whole pathogen to obtain a purified, inert SARS-CoV-2 based on the CN2 strain of the virus. The isolated virus has been grown in Vero cells, which are derived from the kidneys of African green monkeys, and β-propiolactone-inactivated.

Pre-clinical studies of this vaccine indicated established tolerability and immunogenicity profiles. In the murine model, BALB/c, combinations of the alum adjuvant and different doses of the vaccine were introduced to the animals. S and receptor-binding domain (RBD)-specific antibodies reached maximum titers at week 6 post first shot. PiCoVacc was able to enhance S-specific antibody titers by approximately 10 folds compared with sera from recovered COVID-19 patients. Furthermore, vaccination of rhesus macaques with low dose (3 μg) or high dose (6 μg) conferred protective immunity when animals were challenged with natural infection. Indeed, the high dose of PiCoVacc significantly reduced viral numbers in the lung or pharynx 1 week from the infection. S-specific and neutralizing antibodies peaked at week 3 following immunization with both doses, and titers were indifferent from those detected in sera of SARS-CoV-2 recovered patients.^32^

One advantage of this chemically inactivated SARS-CoV-2 vaccine is that it did not trigger pulmonary pathology or disease exacerbation in non-human primates. This point is pivotal for vaccine safety given the immune dysregulation resulted from the virus. PiCoVacc can also elicit neutralizing antibodies against 10 different SARS-CoV-2 strains. In line with data obtained from murine models and non-human primates, phase II clinical evaluation demonstrated that the vaccine is tolerable and immunogenic. Employing 2 different vaccination schedules: 2-week or 4-week intervals between injections with either 3 μg or 6 μg of CoronaVac, the vaccine was efficacious.^33^ These findings supported the progression of phase III clinical testing of the vaccine in the observer-blind, randomized, placebo-controlled studies (NCT04456595, NCT04582344), in Brazil, Turkey, Indonesia, and Chile. Sinovac has recently announced the effectiveness of its vaccine in a media release and it has reported that the vaccine has overall 50.65% efficacy, 83.70% for cases requiring medical interventions, and 100.00% for severe, and fatal cases.^34^

### Protein subunits vaccines

There is always a need to develop well-tolerated and efficacious vaccines. Although live vaccines provoke potent and long-lasting immune responses, they are associated with an increased risk of reversion to virulence. Conversely, inactivated vaccines may be safer but less effective nevertheless. Because of these concerns, subunit vaccines seem to be relevant alternatives to classical vaccine types as they are designed to contain distinctive microbial antigens to induce specific responses in both arms of the immunity.^35^ Subunit vaccines are generally created using recombinant proteins or synthetic peptides. Various subunit vaccines have been developed based on several structural and non-structural viral proteins. A significant drawback in subunit antigens is their weak immunogenicity.^36^ Recent strategies have been under investigation to address this issue by developing self-assembling, robust immunogenic virus-like particles (VLPs), protein polymers, and sub-viral particles.^37,38^ Being able to conserve epitopes of parental B and T cells, and because they are polyvalent in nature, those viral subunits can potentiate humoral and cellular immune responses.^39^ Immunostimulatory substances have been traditionally used to boost waning immunity and maintain long-term protection. Novel adjuvants are being developed, in addition to improving carriers and delivery methods to enhance the immunogenicity of subunit vaccines.^40^ One approach is through engineering fusion proteins in which antigen-adjuvant is introduced as one compound instead of the classic simple mixtures. This subunit-immunostimulator conjugation is pivotal to facilitate the delivery of both substances to the same antigen-presenting cell and ultimately leads to promote the immune system responses.^41^ Furthermore, incorporation of antigens to VLPs and protein polymers creates chimeric combination that activates immense immune events against 2 or more pathogens.^42^

Subunit vaccines can be produced in various expression systems such as viral vectors, bacteria, and yeast and this renders them more suitable for easy, and cost-effective production and distribution than whole organism-containing vaccines.^43^ Subunit vaccines have been recently used to safeguard from emerging, highly pathogenic CoVs. The design of SARS-CoV and MERS-CoV subunit vaccines involves full-length structural S protein, RBD, non-RBD S protein, as well as other non-structural proteins.^44^ The following section overviews current subunit vaccines against COVID-19 and their stages of development.

## NVX-CoV2373

NVX-CoV2373 vaccine development is a collaborative work of Novavax Incorporation and Emergent Biosolutions. NVX-CoV2373 is a subunit vaccine based on a recombinant nanoparticle platform derived from the full-length SARS-CoV-2 trimeric S protein, which is incorporated into the baculovirus expression system and is stabilized in the prefusion conformation. The experimental vaccine exhibits thermostability and can bind to human ACE2 receptor with high affinity.^45^

Early studies indicated that the administration of low dose NVX-CoV2373 along with Matrix-M1, the saponin-based adjuvant, in mice and non-human primates enhanced immune responses. The vaccine stimulated anti-S protein antibodies, and is associated with ACE2 receptor binding blocking. Additionally, NVX-CoV2373 induced SARS-CoV-2 neutralization, helper T cells functions and protection from COVID-19 infection.^45^

Phase I/II of the 2019nCoV-101 (NCT04368988), randomized, placebo-controlled trial assessed the safety and immunogenicity of the vaccine in 131 healthy volunteers. Participants were randomized to receive 5 μg or 25 μg NVX-CoV2373 with or without adjuvant with a 3-week interval between the first and second dose. Reactogenicity profiling of the vaccine demonstrated acceptable safety with only mild symptoms reported. Vaccine/adjuvant regimens stimulated higher levels of anti-S IgG and neutralizing antibodies compared with vaccine alone injections. Likewise, NVX-CoV2373/ Matrix-M1 combination stimulated T-cell responses with an obvious shift towards Th1 cell activity as well as production of TNF-α, IFN-γ, and IL-2.^46^ Consistent with previous reports, a recent study has found that NVX-CoV2373 with its adjuvant has been protective against lower and upper respiratory infection and pulmonary pathology in vaccinated macaques challenged with SARS-CoV-2.^47^ Phase III efficacy trials of NVX-CoV2373/ Matrix-M1 has been launched in the UK with 15000 young and old subjects between 18-84 years of age enrolled in that study. The company stated that the efficacy of the vaccine is 89.3%. In February 2021, European Medicines Agency (EMA) has launched a rolling review of the vaccine and is expected to be approved soon.^48^ The vaccine is cheaper than other alternatives and is easy to transport because it can be stable for 24 hours at room temperature.

## CHO

This vaccine is being developed by Anhui Zhifei Longcom Biopharmaceutical Co. and collaborators in China based on a subunit, recombinant protein platform. The construction of RBD of the MERS-CoV S protein using Chinese hamster ovary (CHO) cells was previously reported. When was added to the adjuvant AddaVax, the protein generated a robust neutralizing antibody response against the MERS-CoV infection in animals.^49^ In a recent report, CHO cells were used to design a stable MERS-CoV RBD dimer with potent immunogenicity and high neutralizing antibody titers in mice challenged with MERS-CoV infection. The vaccine strategy was also applied to SARS and COVID-19, generating even higher neutralizing antibody titers than those achieved in MERS-CoV infection. Pilot studies of the vaccine generated high yields of RBD dimers with the capacity to scale up production, suggesting that this recombinant protein could be applicable for further development.^50^ Phase I/II clinical studies indicated that the protein subunit vaccine is well-tolerated and immunogenic.^51^ The vaccine is currently being assessed for the safety and efficacy in 29000 participants enrolled in international multi-center phase III clinical trials (NCT04646590).

### Nucleic acid vaccines

Since the advent of the concept of genetically engineered DNA vaccines almost 3 decades,^52^ there have been tremendous advances in nucleic acid vaccines, which have opened up the door for a new era in vaccinology. Progress in antigen delivery approaches and effectual transfection have facilitated the efficient uptake of DNA plasmids by cells and thus have improved the robustness of the vaccine’s immunogenicity. It is worthwhile to mention that this kind of vaccine has excellent host-tolerance records. Immunogens dependent on DNA platform have been in development since the 1990s to characterize effective vaccines to target common, emerging and challenging viral infections such as seasonal influenza, HIV, ZIKA, and SARS-CoV.^53,54^ A copious number of clinical studies evaluating DNA-based immunizations in a wide range of diseases, including COVID-19 are run worldwide.

**Figure 2:**
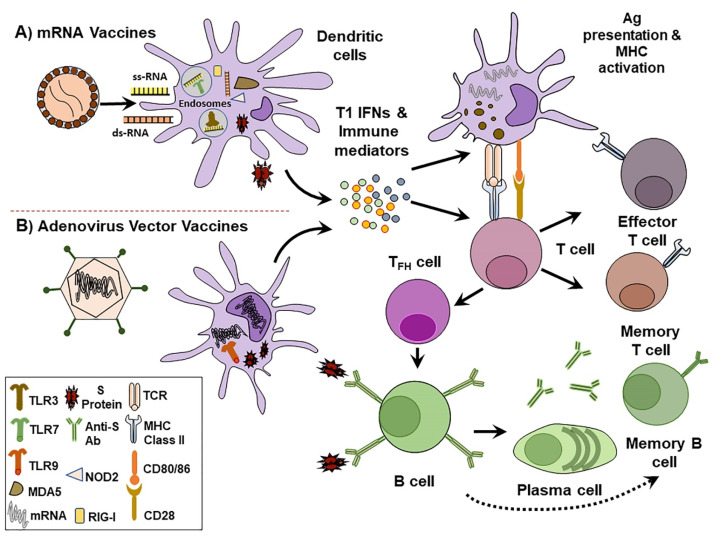
Mechanism of immune system activation by nucleic acid vaccines. A) mRNA vaccines such as Pfizer and Moderna vaccines are composed of LNPs that contain synthetic mRNAs encoding SARS-CoV-2 S protein. B) Adenovirus vectored vaccines such as ChAdOx1 nCoV-19 is a non-replicating vector that expresses a codon-optimized sequence for SARS-CoV-2 S protein. Following injection, vaccines enter local and lymph nodes-containing dendritic cells (DCs) and produce S proteins. This antigen production stimulates pro-inflammatory signals characterized by the secretion of type I interferon (IFN) and other immune mediators. Pathogen recognition receptors (PRRs) within DCs detect nucleic acids. Endosomal Toll-like receptors (TLRs) like TLR3 and TLR7 recognize and bind single-stranded RNA (ss-RNA). Whereas, cytosolic PRRs such as Nucleotide-binding oligomerization domain-containing protein 2 (NOD2), retinoic acid-inducible gene I (RIG-I) and melanoma differentiation-associated protein 5 (MDA5) bind double-stranded RNA (ds-RNA). Adenoviral associated ds-DNA is mainly distinguished by TLR9. As antigen presenting cells, DCs display S proteins to promote major histocompatibility complex (MHC) and induce T cell specific activation. Upon stimulation, T cells differentiate into effector cells to engage helper and cytotoxic T cells mediated immune responses. Follicular helper T cells (TFH) direct S protein activated B cells to differentiate into plasma cells and produce anti-S protein antibodies. Circulating antibodies against S proteins protect from SARS-CoV-2 infection.

There is a growing interest in RNA-employing approaches for vaccine development for prophylaxis from illnesses. RNA and its derivatives like mRNA hold the potential to replace conventional strategies of vaccinations against newly discovered microbes.^55^ This is owing to their safety since they do not contain infectious parts of the pathogen and its immunogenicity can be endogenously regulated. Add to that, the non-stability of mRNA can be “fine-tuned” and modified therefore the immunogenicity is enhanced.^56,57^ mRNA does not only activate humoral immunity but can also substantially drive prolong T cell-mediated responses.^58^ Another beneficial aspect of mRNA vaccines is their flexibility for use with varying carriers, and capacity to be cost-effective, and scalable for mass production and rapid deployment.^59^ Structure-guided antigenic design, mRNA optimized sequences, improved delivery by lipid nanoparticles (LNPs), and prior knowledge of beta CoVs have allowed the application of mRNA technologies to shorten the period required for SARS-CoV-2 vaccine development. Here, we explore the most promising COVID-19 vaccines based on nucleic acid platforms presently under clinical assessment. The mechanism of activating immune system by nucleic acid vaccines are elucidated in (Figure 2).

## University of Oxford Vaccine (ChAdOx1 nCoV-19)

The ChAdOx1 nCoV-19 (AZD1222) is a viral vectored vaccine developed by the University of Oxford. ChAdOx1 is based on Chimpanzee adenoviral vector,^60^ and was designed to express a codon-optimized sequence for SARS-CoV-2 S protein. The ChAdOx1 construct is made up of a non-replicating adenovirus vector, containing a full-length SARS-CoV-2 S glycoprotein sequence in addition to a tissue plasminogen activator (TPA) leader sequence.

The candidate vial-vectored vaccine ChAdOx1 encoding MERS-CoV S protein was previously reported to elicit cellular and humoral immune responses against MERS-CoV infection, with reasonable safety and tolerability.^61^ A single dose of the vaccine has been able to confer protective immunity against MERS-CoV and reduce disease severity in rhesus macaques.^62^ Based on these reports, ChAdOx1 was also used to design SARS-CoV-2 vaccine. ChAdOx1 nCoV-19 was immunogenic in mouse models and rhesus macaques, inducing robust humoral and cellular-mediated immune responses. In macaques, ChAdOx1 nCoV-19 substantially reduced SARS-CoV-2 viral load with the absence of pneumonia in the lungs of vaccinated non-human primates, supporting the efficacy of the vaccine.^63^

Earlier reports on ChAdOx1 nCoV-19 showed that it is generally safe and well-tolerated in people who were given the vaccine. Expected side effects such as fever and headache were reported and they were resolved by the administration of prophylactic paracetamol. Phase I/II (NCT04324606) single-blinded, randomized controlled study tested the reactogenicity and immunogenicity of the candidate vaccine in 1077 participants. They were divided into 2 groups to receive either ChAdOx1 or the comparator (MenACWY) as a control vaccine. Anti-S protein T-cell immune responses reached maximum levels 2 weeks post-vaccination. Furthermore, humoral responses peaked 4 weeks after prime dose in more than 90% of participants, and demonstrated a 4-fold increase in all individuals progressed from prime to boosting dose.^64^ These findings led to the progression into late-stage phase II/III clinical trials in different countries. More recently, phase II/III findings have demonstrated that the ChAdOx1 vaccine has good tolerability in older adults compared with younger participants and has similar immunogenicity profiles across all tested age groups following the boost dose.^65^ Moreover, data analyses from 20 000 participants enrolled in phase II/III randomized, placebo-controlled trial of ChAdOx1 (NCT04400838) in the UK, South Africa, and Brazil revealed good safety and efficacy profiles.^66^ The vaccine has been recently approved for human use in many countries. Importantly, it can be stored at 2–8°C, making it feasible for global distribution.

## INO-4800 (Inovio Pharmaceuticals)

It is a DNA vaccine targeting SARS-CoV-2 surface protein, which is being developed based on a previously synthesized DNA vaccine against MERS-CoV.^67^ The strategy of vaccine design utilizes sequence homology between S proteins of beta CoVs. Since these S proteins share similar host receptor, ACE2, they are essential targets for vaccine development.^68^

The INO-4800 construct contains an IgE leader sequence incorporated into an optimized SARS-CoV-2 S protein and digested with endonucleases BamHI and XhoI. The DNA sequence was then cloned into the plasmid pGX0001 and controlled with a human cytomegalovirus promoter. Using the smart delivery device, CELLECTRA® 2000, a single dose of INO-4800 generated detectable IgG antibodies against SARS-CoV-2 S protein and its RBD in sera of immunized mice 2 weeks post-vaccination. Similar to findings observed in mice, intradermal delivery of 100 µg of INO-4800 in guinea pigs elicited humoral immune response to S proteins at day 14 of immunization. Because ACE2 is the entry point of SARS-CoV-2 to the infected cells, inhibition of SARS-CoV-2 S protein-ACE2 binding is crucial for disease prevention. INO-4800-induced antibodies inhibited the S protein-ACE2 interaction in the active vaccine group as compared with the control mice through competing with ACE2 binding sites of the S protein.^69^ Immunogenicity and efficacy assessment of INO-4800 in rhesus macaques demonstrated relatively long-term protection against SARS-CoV-2 challenge by inducing anti-S protein cellular mediated responses several months following vaccination.^70^ In phase I clinical evaluation, intradermal administration of either a 1.0 mg or a 2.0 mg of INO-4800 was found to be 100% immunogenic.^71^ While phase I (NCT04336410) was begun in April 2020, phases II/III of clinical trials are still ongoing. The phase II part of the placebo-controlled clinical trial evaluates the safety, tolerability, and immunogenicity of the vaccine in a 2-dose protocol (1.0 mg or 2.0 mg) to select the safest immunogenic dose for the age groups included in the study. Phase III efficacy assessment will focus on high risk of infection in individuals (18-65 years and older). The DNA vaccine is stable at room temperature and does not require freezing, which makes it a better option in terms of logistics and transport.

## Ad5-nCoV (CanSino Biologics)

The Ad5-nCoV is a non-replicating adenovirus vectored vaccine expressing optimized full-length SARS-CoV-2 S glycoprotein with TPA signal peptide gene. Ad5-nCoV clinical trial is the first to test a vaccine for COVID-19 in China. In phase I clinical studies, 108 adult subjects (18-60 years) were screened for any adverse reactions the vaccine would elicit, and it was overall tolerable with mild to moderate symptoms. The recombinant vaccine activated significant T-cell responses to S protein 2 weeks after administration and humoral responses 4 weeks after the first injection.^72^

Over 500 participants were enrolled in the randomized, double-blind, placebo-controlled phase II (NCT04341389) clinical trial to receive either a low dose (5*10^10^ viral particles), a high dose (1*10^11^ viral particles) or a placebo. Both vaccine doses generated comparable SARS-CoV-2 neutralizing antibody titers 4 weeks after immunization. Antibody responses to RBD were detectable at day 14 post-vaccination with both low and high doses and scored nearly a 7-fold induction at day 28. At the same time point, positive cellular immune responses were observed in most volunteers in low and high-dose groups.^73^

One drawback of this vaccine is the baseline Ad5 immune responses detected in many older participants and could interfere with stimulating robust humoral responses. Additionally, the reported findings covered a short duration and this would question the vaccine’s ability to generate and maintain long-term immunity. Moreover, we do not have information regarding how shall the vaccine perform when immunized participants are challenged with COVID-19 infection, a question that should be answered in phase III trials.

The global, multi-center phase III (NCT04526990) clinical trial is ongoing in several countries, including Russia, Pakistan, and Saudi Arabia, and is planned to enroll 40 000 individuals, 18 years and older. This randomized, double-blind and placebo-controlled study assesses the efficacy, reactogenicity and immunogenicity of the experimental vaccine, and is expected to be concluded by the end of 2021. More recently, China has approved rolling out this vaccine before the completion of clinical studies and is expected for mass distribution soon. Unlike many other vaccines, Ad5-nCoV can be effective and may trigger an immune response in one single dose.

## Ad26.COV2.S (JNJ-78436735)

Ad26 is a recombinant replication-deficient adenovirus type 26 vector (Ad26). It is based on previous viral-vectored vaccine designs that were tested on several viral infections such as HIV, Ebola, and Respiratory syncytial virus.^74,75^ It displayed acceptable tolerability and immunogenicity in humans by stimulating binding and neutralizing antibody activities, and a Th1-shifted immune response in both animals and humans.

Using different variants of the S protein, researchers adapted previously described structural designs to optimize SARS-CoV-2 S glycoprotein to stabilize it in the prefusion conformation. The modification involved site-specific mutations in the furin cleavage at the S1/S2 subunit boundaries, along with 2 substitutions in proline residues. As a result, neutralizing antibodies were increased compared with non-neutralizing antibody binding, indicative of S glycoprotein stabilization and improved immunogenicity of the vaccine. As far as cellular immunity is concerned, the optimized Ad26 vaccine design demonstrated Th1 bias. Mice were vaccinated with 10^8^ or 10^10^ of native or optimized Ad26 produced IFN-γ and the individual ratios of IFN-γ secretion to IL-4, IL-5 or IL-10 were high after administration of optimized Ad26, suggestive for Th1 polarization.^76^

The double-blind, placebo-controlled (NCT04505722) phase III ENSEMBLE trial is resumed after temporary suspension to investigate serious adverse event experienced by one of its participants. It enrolled 60,000 healthy adult subjects (18 years and older) in addition to individuals at risk of developing severe symptoms to assess the safety and efficacy of Ad26.COV2.S in the prevention of positive COVID-19 cases. The interim report of phase I/II trials was published in the New England Journal of Medicine where the vaccine showed favorable safety and immunogenicity profiles.^77^ In a more recent statement, the US Food and Drug Administration (FDA) has approved the vaccine for emergency use in 18 years adults and older individuals.^78^

## Gam-COVID-Vac (Sputnik V)

The Gamaleya National Center’s vaccine is a heterologous, recombinant adenoviral vectored-based vaccine against SARS-COV-2, comprised of 2 adenovirus vectors type 26 (Ad26) and type 5 (Ad5), carrying the S glycoprotein gene.

A total of 76 healthy adult volunteers were enrolled in phase I/II clinical trials (NCT04436471 and NCT04437875). The primary outcome indicated acceptable reactogenicity with only mild adverse events reported and a strong immunogenicity profile. Implementing a prime-boost vaccination regimen, Gam-COVID-Vac produced robust humoral and cellular mediated immune responses in 100% of healthy subjects. Antibody responses were higher than those detected in human sera from individuals who have recovered from SARS-COV-2 infection.^79^

As the first registered and distributed COVID-19 vaccine in Russia, Sputnik V has triggered discussions regarding the accuracy of findings of the early-phase clinical studies, hence the safety and effectiveness of the vaccine.^80^ In fact, there are concerns about probable duplication of results and seemingly repetitive data patterns in the original paper published in *The Lancet*. More importantly, the vaccine was released without progressing to phase III clinical trials where rigorous analysis of the safety and efficacy is extensively conducted on a large number of participants. Currently, EMA is investigating the efficacy of Sputnik V in a rolling review to see if the vaccine complies with the European Union standards of safety and effectiveness.^81^

## mRNA-1273

This nucleic acid-based vaccine is co-developed by Moderna Incorporation and National Institute of Allergy and Infectious Diseases. The vaccine is composed of a novel LNP that contains a synthetic mRNA strand encoding full-length, prefusion stabilized SARS-CoV-2 S protein trimer (S-2P). It was designed to harbor 2 proline substitutions in the S2 subunit of the S protein.

The open-label, dose-escalated phase I clinical trial (NCT04283461) was conducted on 45 healthy volunteers (18-55 years), and participants were allocated to 3 groups to receive either 25 μg, 100 μg, or 250 μg of the vaccine. The increased titers of S protein neutralizing antibodies in sera of tested individuals positively correlated with the higher doses of the vaccine. As far as safety was concerned, mild to moderate symptoms were detected in participants and systemic adverse effects were corresponded with the 250 μg dose.^82^ The phase I clinical trial was expanded to include 40 older individuals assigned to 2 groups: 56-70 years and 71 years and older. Participants were enrolled in a 2-dose vaccination regimen; 25 μg and 100 μg. Data suggested that the immune activity was dose-dependent where the highest concentration elicited increased levels of humoral and T-cell dependent responses in a similar trend to that of younger adults.^83^ Similarly, there was a dose-dependent induction of neutralizing and binding antibodies to S-2P after vaccine administration in rhesus macaques. It was noted that antibody levels surpassed those detected in humans convalescent sera. Regarding cellular responses, induced Th1 cells were detected in half of the immunized animals 4 weeks after second vaccination with 10 μg, and in all vaccinated macaques administered with 100 μg of mRNA-1273. Conversely, both vaccine doses generated low or undetectable Th2 cells responses. Additionally, lung tissues from macaque animals treated with 100 μg of mRNA-1273 and challenged with the virus did not show significant inflammation and viral RNA was undetectable in these lung sections.^84^

As of October 22, 2020, 30 000 participants were involved in phase III clinical study in the US, and approximately 26 000 individuals received their second vaccination. Results of this study indicated very high protection (94.1%) against COVID-19.^85^ More importantly, mRNA-1273 was the second nucleic acid-based vaccine to be authorized by FDA for emergency use and has received approval in many countries around the world.

## BNT162 (COMIRNATY®)

The development of BNT162 vaccines is a part of the “Lightspeed” project, a collaboration between BioNTech SE, Pfizer, and Fosun Pharma, that was carried out in Germany (NCT04380701), the US (NCT04368728), and China. BNT162 vaccines are a group of 4 mRNA candidate vaccines (BNT162a1, BNT162b1, BNT162b2, and BNT162c2) formulated in LNP to target large spike sequence and optimized RBD of SARS-CoV-2 S protein, for the prevention of COVID-19 disease.^86^

The trial started in Germany in April 2020 and is consisted of 2 parts. The first part was for dose testing and optimization, which included dose escalation and de-escalation. The second part was dedicated to expand cohorts and evaluate selected doses. All candidate vaccines were administered using a two-dose (prime and boost) regimen, except for BNT162c2 which was given using a single dose regimen as well.

BNT162b1 is a modified, nucleoside-mRNA vaccine combined with LNP formulation that encodes trimerized SARS-CoV-2 S protein RBD. The preliminary data of the safety, reactogenicity and immunogenicity of the phase I/II placebo-controlled clinical trial (NCT04368728) indicated that BNT162b1 is generally safe and well-tolerated. Participants received 2 doses of 10 μg, 30 μg, or 100 μg of the candidate vaccine with a 3-week interval between doses. Serum RBD-binding and neutralizing antibody levels followed a dose-dependent trend with a marked increase in titers after the boost dose. The mean of neutralizing antibody titers exceeded that of convalescent human sera from SAR-CoV-2 positive cases.^87^ Furthermore, 50 μg of BNT162b1 induced potent T cell-mediated responses, including IFN-γ and S protein neutralizing antibody concentrations were more than those detected in SARS-CoV-2 infected convalescent human sera.^88^

In cohort expansion, placebo-controlled phase I trial, 195 healthy participants aged 18 to 55 years (younger group) and 65 to 85 years (older group) were assigned to receive either BNT162b1 or BNT162b2, or placebo. In terms of vaccine safety assessment, BNT162b2 was safer than BNT162b1 in the older cohorts, generating less severe systemic adverse events. There was a similar trend between the two vaccine candidates in inducing dose-dependent neutralizing antibody activities among younger and older adults. Antibody responses to BNT162b1 or BNT162b2 (10 μg to 30 μg), however, were generally lower in older adults than in younger participants. The second dose of the vaccine augmented antibody levels and the highest titers were detected on day 7 and day 14 following administration.

Phase II/III clinical studies showed that the 2-dose regimen (30 μg) of BNT162b2 stimulated robust immune responses and conferred 95% protection against SARS-CoV-2.^89^ Taken together, BNT162b2 exhibited excellent protection and has been approved now for use in about 65 countries. Phase IV trial (NCT04760132) is underway in Denmark to assess the long-term effectiveness and safety of the vaccine.

## CVnCoV (CV07050101)

CVnCoV is a prophylactic vaccine against COVID-19 established by CureVac with collaboration from Coalition for Epidemic Preparedness Innovations. This vaccine is engineered using LNP formulation with mRNA based platform to encode full-length SARS-CoV-2 S protein. To stabilize the protein in the prefusion conformation, two proline residue substitutions (S-2P) were generated similar to previously synthesized SARS-CoV and MERS-CoV immunogens.^90^ Pre-clinical studies in mice and hamsters suggested that CVnCoV was capable of inducing innate and adaptive immune responses. The mRNA vaccine initiated robust humoral responses reflected by increased SARS-CoV-2 neutralizing antibody titers. Additionally, mice immunized with CVnCoV exhibited potent cellular immune responses balanced for both CD4+ and CD8+ T cells. Collectively, in the absence of increased CVnCoV-related viral numbers in the respiratory tract and exacerbated inflammation in the lung, the tested vaccine displays an acceptable safety profile.^91^

Phase 1 (NCT04449276) clinical trial of CVnCoV started in late June 2020 at various clinical centers in Germany and Belgium where dose-range testing and vaccine safety were evaluated. Younger and older participants were recruited to the phase 2a (NCT04515147) clinical study in Panama and Peru for dose selection and confirmation. In this ongoing trial, subjects were randomly allocated to receive different doses of the investigational vaccine or the active comparator hepatitis A or pneumococcal vaccines. Previous data suggested 12 µg of CVnCoV to proceed for phase 2b/3 (NCT04652102) clinical study, which was launched in December 2020. On February 12, 2021, CureVac has applied for a rolling submission to EMA to review the efficacy of the vaccine, and if successful, it may be available soon. Of note, CureVac’s vaccine has one vital advantage over few other vaccines such as those developed by Moderna and Pfizer, characterized by its stability at 5 °C.

## LNP-nCoVsaRNA

The vaccine was designed by Imperial College London and co-funded by Medical Research Council and UK Research and Innovation. It is another vaccine that utilizes an LNP formulation platform to pack the self-amplifying RNA (saRNA) that encodes a stabilized SARS-CoV-2 S protein. The saRNA technology means the vaccination may be achieved with a low dose compared to mRNA vaccines. The immunogenicity profiling of LNP-nCoVsaRNA in a murine model indicated robust humoral and cellular immune responses, which were higher than those generated by SARS-CoV-2 natural infection. In the pre-clinical studies, mice were exposed to 2 injections, 4 weeks apart, of varying doses (0.01 to 10 μg) of LNP-nCoVsaRNA. All vaccine concentrations, including the minimum dose (0.01 μg) stimulated higher SARS-CoV-2 IgG titers than those detected in COVID-19 recovered patients. Virus-specific IgG levels were dose-responsive, and were correlated with SARS-CoV-2 neutralization. Of importance, there was a significant induction in the secretion of a panel of cytokines, including IL-4, IL-6, IFN-γ, and TNF-α in LNP-nCoVsaRNA vaccinated mice.^92^ There is an ongoing randomized, controlled phase I/II clinical trial (COVAC1) to evaluate the safety and immunogenicity of the vaccine in young and old adults, and possibly proceed to the efficacy testing in phase III studies.^93^

While many clinical studies dedicated for COVID-19 vaccines were concluded, others are still ongoing. The main vaccines against SARS-CoV2 are presented in (Table 1).

**Table 1:** List of the most important COVID-19 vaccines in clinical trials

**Vaccine/** **developer**	**Type** **(platform)**	**Study objectives**	**Dose & route of administration**	**Clinical Stage**			
				**Phase 1**	**Phase 1/2**	**Phase 2**	**Phase 2/3** **Phase 3**
ChAdOx1 University of Oxford/AstraZeneca	Replication-deficient Viral Vector	Examine the safety, efficacy, and immunogenicity of AZD1222, a non-replicating ChAdOx1 vector vaccine, for the prevention of COVID-19	2 injections At days 0 & 28 IM Low dose (2.2 × 10^10^) virus particles and standard dose (3·5–6·5 × 10^10^) virus particles in adults of different age groups		NCT04324606 NCT04568031 ^64^		NCT04400838 NCT04516746 NCT04540393 NCT04536051 ^65^
INO-4800 Inovio Pharmaceuticals	DNA vaccine	To test the safety, reactogenicity, and immunogenicity profiles of INO-4800 injected intradermally followed by electroporation (EP) by CELLECTRA® 2000 device in healthy adult volunteers aged 19 to 64 years	INO-4800 1mg or 2mg/dose + EP by CELLECTRA® 2000 (at Day 0 and 28)	NCT04336410 ^70,71^	NCT04447781		
mRNA-1273 Moderna	LNP-encapsulated mRNA	To evaluate the safety, efficacy, and immunogenicity of the experimental vaccine mRNA-1273 for the prevention of COVID-19 for up to 2 years after the boost dose.	100 µg Administered at days 0 & 29 IM	NCT04283461 ^82,83^		NCT04405076	NCT04470427 ^85^
BNT162 vaccines BioNtech & Pfizer	3 LNP-mRNAs	Assessment of the safety, tolerability, and immunogenicity of 2 SARS CoV-2 RNA vaccine candidates against COVID- 19 and the efficacy of another candidate (BNT162b2).	30 μg of BNT162b2 Prime-boost regimen administered IM	^94^	NCT04537949 NCT04588480 ^87,95^		NCT04368728 ^89^
NVX-CoV2373 Novavax	Protein Subunit	This study is designed to evaluate the safety, effectiveness, and immune response of the COVID-19 vaccine (SARS-CoV-2 rS) with Matrix-M1 adjuvant in adults ≥ 18 years	5 μg SARS-CoV-2 rS + 50 μg Matrix-M1 adjuvant (co-formulated), given on days 0 & 21.		NCT04368988 ^46^	NCT04533399	NCT04611802
CVnCoV CureVac	LNP-mRNA	Randomized, controlled trial to assess the safety, and immunogenicity of CVnCoV in adults >60 and 18-60 years.	12 μg 2 doses injected IM on days 1 & 29	NCT04449276		NCT04515147	
Ad5-nCoV CanSino Biologics	Recombinant, non-replicating adenovirus vectored vaccine	Double-blind, placebo-controlled trial to evaluate the safety and efficacy of Ad5-nCoV in healthy subjects ≥ 18 years	Single dose contains (5*10^10^) viral particles injected IM	NCT04313127 NCT04552366 ^72^		NCT04566770 NCT04341389 ^73^	NCT04526990 NCT04540419
Ad26.COV2.S Janssen Vaccines & Prevention	Non-replicating Adenovirus Vector Type 26	A randomized, double-blind, placebo-controlled clinical trial to evaluate the efficacy and safety of Ad26.COV2.S for the long-term protection and prevention of COVID-19 in adults ≥ 18 years	2 doses injected IM on days 1 & 57 or Single-dose contains (5*10^10^) viral particles administered IM On day 1	NCT04509947 ^76^	NCT04436276 ^77^	NCT04535453	NCT04505722 NCT04614948
Gam-COVID-Vac Gamaleya Research Institute	Heterologous, non-replicating Adenovirus vector (rAd26-S+rAd5-S)	Evaluating the efficacy, immunogenicity, and safety of the Gam-COVID-Vac prophylactic vaccine against COVID-19 in adults ≥18 years.	Gam-COVID-Vac combined vector vaccine, 0,5ml/dose+0,5 ml/dose prime-boost IM injections on days 1 (component I rAd26-S) and 21(component II rAd5-S)		NCT04436471 NCT04437875 ^79^	NCT04587219	NCT04530396 NCT04564716
Covaxin (BBV152) Bharat Biotech	Whole-virion Inactivated	Randomized, double-blind, placebo-controlled clinical study to evaluate the efficacy, safety, and immunogenicity of BBV152 to prevent COVID-19 for up to 1 year following the boost dose, in adults ≥18 years.	Administered as a 2-dose regimen IM injections, 28 days apart	^30,31^	CTRI/2020/07/026300 CTRI/2020/09/027674 NCT04471519		CTRI/2020/11/028976 NCT04641481
Beijing & Wuhan Institutes of Biological Products/Sinopharm vaccines	Inactivated vaccines	The primary objective of this randomized, double-blind, placebo-controlled study is to assess the protective impact and efficacy of inactivated SARS CoV-2 vaccine (Vero Cell) for preventing SARS CoV-2 related diseases in healthy adults ≥18 years.	2 doses injected IM on days 0 & 21		ChiCTR2000031809 ^27,29^		NCT04560881 NCT04612972

### Others

Apart from prophylactic vaccines that specifically target SARS-COV-2 or its subunits, different approaches of protection from the virus are in assessment. These strategies are intended to alleviate the severity of the disease in high-risk groups such as healthcare workers. Health professionals are more prone to COVID-19 because of the nature of their work that necessitates physical contact with patients, thereby increasing the possibility of contracting the infection.^96^ This could endanger the safety of health personnel who may turn out to be a source of transmitting the infection and exerting a heavy burden on the health system by interfering with the continuous patient care needed to tackle the disease. To prevent this, repurposing of available vaccines was adopted to protect medical and health staff. For example, the tuberculosis Bacillus Calmette-Guérin (BCG) vaccine, was proposed to boost the immunity in the more susceptible persons in the frontline of fighting COVID-19.^97^ Of note, countries that adopted BCG vaccination reported low virus infectivity and mortality rates compared with states that do not apply this vaccination policy.^98^ BCG was reported to offer off-target protection against non-mycobacterial infections,^99^ and recombinant BCG strains were used as a platform to express antigens of various infectious pathogens. This antigen delivering strategy could be applied to SARS-COV-2 to activate innate and, to some extent, virus-specific immune responses. It was hypothesized that the “trained immunity”, an augmented immune response to a particular pathogen following vaccination or exposure to a different infectious agent, could be a beneficial strategy in combating COVID-19.^100^ Trained innate immune responses are associated with elevated levels of pro-inflammatory cytokines and are thought to be mediated by histone dependent epigenetic reprogramming and chromatin remodeling in immune cells.^101^ This could explain the protective impact of BCG on unrelated infections. Several ongoing clinical trials are evaluating the potential protective effects of BCG against SARS-COV-2 in healthcare professionals in the USA, Europe, and Australia (Table 2).^102^ Based on this concept, other live attenuated vaccines such as polio and Measles-Mumps-Rubella (MMR) vaccines were suggested to mitigate the SARS-COV-2 sequelae.^103^ One study has found that MMR vaccination afforded protection to COVID-19 patients and they were presented with mild symptoms.^104^ Another report suggested the use of MMR vaccine as a preventive measure against COVID-19 associated inflammation and sepsis.^105^ However, this data warrants a thorough clinical investigation into the advantages of using non-specific vaccines in ameliorating COVID-19 severity.

**Table 2:** Non-COVID-19 specific vaccines. This table lists live-attenuated vaccines that enhance trained innate immune responses against SARS-CoV-2

**Vaccine/** **Developer**	**Type** **(platform)**	**Study objectives**	**Clinical stage**	**Dose & route of administration**
BCG (TASK Applied Science)	Live attenuated	Randomized, placebo-controlled. Reducing SARS-CoV-2 associated morbidity and mortality in healthcare workers by enhancing BCG dependent off-target immune responses	NCT04379336 (phase 3)	Intradermally One dose (0.1 mL**)**
BCG (Radboud University)	Live attenuated	Randomized, placebo-controlled study to evaluate: 1. BCG ability to minimize hospital admission. 2. BCG efficacy to reduce the severity of SARS-CoV-2 infection in elderly subjects (60 years or older).	NCT04417335 (phase 4)	Intracutaneous administration
BCG (Tuberculosis Research Centre, India)	Live-attenuated	To assess the effectiveness of BCG vaccine in preventing COVID-19 related morbidity and mortality in elderly persons (60-80) years living in COVID-19 affected areas in India.	NCT04475302 (phase 3)	A single dose of BCG vaccine (0.1 mL), injected intradermally
BCG (University of Campinas, Brazil)	Live-attenuated	Randomized, double-blind, placebo-controlled. To study the impact of previous or current BCG vaccination (boost with intradermal vaccine) on: 1. Clinical evolution of COVID-19 2. Elimination of SARS-CoV-2 3. Seroconversion rate and levels of (anti-SARS-CoV-2 IgA, IgM, and IgG).	NCT04369794 Phase 4	A single dose of BCG vaccine (0.1 mL), administered intradermally
BCG Hellenic Institute for the Study of Sepsis	Live-attenuated	A randomized, double-blind, placebo-controlled clinical trial. Evaluation of efficacy and safety of BCG Vaccine in the prevention of COVID-19 in susceptible participants (50 years) or older.	NCT04414267 Phase 4	A single dose BCG vaccine (0.1 mL), intradermal injection
BCG UMC Utrecht	Live-attenuated	A randomized, placebo-controlled clinical trial. To reduce absenteeism among healthcare professionals with direct patient contacts throughout the epidemic stage of Covid-19.	NCT04328441 Phase 3	A single dose BCG vaccine (0.1 mL), intradermal injection
BCG UMC Utrecht	Live-attenuated	A randomized, placebo-controlled clinical trial. To determine the effect of BCG vaccine on the incidence of respiratory infections or COVID-19 in vulnerable elderly (60 years) or older.	NCT04537663 Phase 4	A single dose BCG vaccine (0.1 mL), intradermal injection
BCG Hospital Universitario Dr. Jose E. Gonzalez	Live-attenuated	A randomized double-blind clinical trial. To evaluate efficacy and safety of BCG vaccine in prevention of COVID-19 among healthcare workers.	NCT04461379 Phase 3	BCG vaccine 0.1 mL given intradermally
BCG Murdoch Children’s Research Institute	Live-attenuated	A randomized double-blind, clinical trial. To determine the ability of BCG vaccination in reducing the incidence and severity of COVID-19.	NCT04327206 Phase 3	BCG vaccine (0.1 mL) injected intradermally
BCG Assistance Publique - Hôpitaux de Paris	Live-attenuated	A randomized, placebo-controlled trial to test the efficacy of BCG vaccination in the prevention of COVID-19 through the enhancing of innate immunity in healthcare workers.	NCT04384549 Phase 3	A single dose (0.1 mL) of BCG administered intradermally
BCG Bandim Health Project	Live-attenuated	A randomized, placebo-controlled trial assessing BCG vaccine to activate non-specific protection of healthcare workers throughout the COVID-19 pandemic.	NCT04373291 Phase 3	A single dose of BCG vaccine (0.1 mL), intradermal injection
BCG Texas A&M University	Live-attenuated	A randomized, placebo-controlled trial to protect healthcare workers by stimulating trained immune responses	NCT04348370 Phase 4	A single dose of 0.1 mL of BCG given intradermally
BCG University Health Network, Toronto	Live recombinant	A randomized, double-blind, placebo-controlled to assess the efficacy and safety of VPM1002 in reducing the incidence of SARS-CoV-2 infection and COVID-19 Severity.	NCT04439045 Phase 3	VPM1002 (Recombinant BCG) vaccine (0.1 mL) injected intradermally
BCG Vakzine Projekt Management GmbH	Live recombinant	A double-blind, randomized, placebo-controlled clinical trial to evaluate: 1- The efficacy and safety of recombinant BCG (VPM1002) in reducing absenteeism of healthcare professionals due to SARS-CoV-2 Pandemic by modulating the immune system responses. 2- Reducing hospital admissions and/or severe respiratory infections due to COVID-19 in elderly individuals (60 years) or older.	NCT04387409 NCT04435379 Phase 3	VPM1002 (0.1 mL) administered intradermally
MMR (Louisiana State University Health Sciences Center in New Orleans)	Live-attenuated	A randomized, placebo-controlled clinical trial to assess whether vaccination with MMR (measles mumps rubella; Merck) can elicit non-specific trained immunity that can prevent or ameliorate COVID-19 associated inflammation and sepsis in adults at very high risk for contracting SARS-CoV-2 infection (such as healthcare workers, first responders).	NCT04475081 Phase 3	-
MMRII^®^ (Washington University School of Medicine	Live-attenuated	A randomized, placebo-controlled clinical trial evaluating the effectiveness of MMR vaccine in alleviating COVID-19 disease in healthcare workers.	NCT04333732 Phase 3	-
Polio vaccine NeuroActiva, Inc.	Live-attenuated	A randomized, double-blind, placebo-controlled clinical study to assess the safety and efficacy of oral polio vaccine and the drug NA-831 as a therapeutic agent and prophylactic vaccine to prevent early onset of Covid-19	NCT04540185 Phase 3	Polio vaccine bivalent OPV (GSK), (0.1 mL) given orally NA-831 (30 mg) as a capsule administered orally
Polio vaccine Bandim Health Project	Live-attenuated	A randomized trial to assess the impact of oral polio vaccine in persons above 50 years. To test if the vaccine reduces the combined risk of morbidity or mortality by at least 28% over 6 months.	NCT04445428 Phase 4	Polio vaccine bivalent OPV (GSK), (0.1 mL) given orally

To date, there is no approved, efficacious therapeutic solution for SARS-COV-2 associated pathologies, but various avenues have been linked with the treatment and/or prophylaxis of COVID-19, involving passive immunity. People who recover from infectious diseases often produce high titers of antibodies that neutralize the effect of the underlying pathogens. This antibody response usually provides a timeframe immunity to protect from re-infection. Convalescent plasma (CP) has long been used in the treatment of infections, and in particular during pandemics.^106^ Recently, FDA has approved the CP use for treating critically ill COVID-19 patients,^107^ but with the conflicting results reported regarding its benefit,^108,109^ it is unclear whether the transfusion of CP to COVID-19 patients is a safe and effective clinical practice. This is particularly important in the absence of data from large-scale clinical studies that support the effectiveness of CP as immunotherapy in COVID-19 patients. It is also of significance to identify the optimal dose of CP that contains high-level neutralizing antibodies which are adequate to trigger anti-viral immune responses and mitigate viremia.^110^ Currently, a large number of studies are underway to clinically assess the use of CP as a therapeutic for COVID-19, and there are approximately 25 trials are in phase III or IV and are anticipated to conclude soon (Table 2).

## Concluding remarks

SARS-COV-2 has emerged as an unprecedented global health concern that has had a significant impact on various aspects of our lives, including economic activities, education, and well-being. Since it has been declared as a global pandemic, worldwide efforts have been teamed up to confine the virus and control its spread. From the clinical perspective, there is no distinct approved treatment for COVID-19 to date. Despite the tireless work the scientists and biotechnology ventures across the globe are doing to develop tolerable and efficacious anti-COVID-19 vaccines, the puzzle of the overwhelming pandemic has yet to be solved. Vaccine derivation is a quite tedious and labor-intensive process that involves pre-clinical and three-phase clinical studies, which normally take several years to complete. To overcome this lengthy process, it has been imperative to bypass stages without compromising the integrity of the research and quality control analyses. We have been fortunate to learn from past lessons in facing acute respiratory outbreaks to accelerate vaccine development for COVID-19. Sequence similarities among SARS-COV, MERS-CoV, and SARS-COV-2, the knowledge of antigen epitopes of the target virus, mapping immune responses to SARS-COV-2 are essential for vaccine design. In addition, utilizing the already known techniques in genetic engineering to manipulate nucleic acids, viral subunits, or whole pathogen inactivation have streamlined the vaccine creation. It is noteworthy to mention that data review and regulatory approval have been fast-tracked.

Practically, a thoughtful understanding of the antigenicity characteristics of a vaccine, and the antigen delivery strategies is a crucial step in its development. Vaccine formulation, route of administration, added adjuvants, as well as dose optimization, and vaccination schedules are all critical contributing factors to the effectiveness of any vaccine. The capacity of producing efficacious immunogens is also determined by their thermostability.^111^ Ideally, vaccines with long shelf-life and the possibility of storage at ambient temperatures are preferred to those that require freezing as this adds to the total production cost of vaccines.^112^ Regardless of licensure, one major obstacle for a feasible vaccine would be how to scale up manufacturing to secure a rapid mass production of billions of doses needed to meet global demand. From the logistics point of view, smooth distribution of a licensed vaccine should be taken into consideration in the light of pre-arranged immunization supply chains that wealthy nations have signed with manufacturers to ensure sufficient injections to their people. This could impose challenges for both suppliers and healthcare authorities in developing countries that may not have adequate vaccine coverage,^113^ unless the COVAX initiative is effectively applied.

Many vaccines are in different phases of clinical development, but only a handful has progressed to late stages nonetheless. At the time of preparing this manuscript, 12 vaccines have been approved for human use in various countries (Table 3).

**Table 3:** List of approved COVID-19 vaccines

**Vaccine (developer)**	**Date of approval**	**Countries approved in**
CanSino Biologics	June 2020	Mexico, China (military use), Pakistan
Sinovac Biotech	July 2020	Azerbaijan, Bolivia, Brazil, Cambodia, China, Chile, Colombia, Hong Kong, Indonesia, Laos, Mexico, Thailand, Turkey, Philippines, Uruguay
Sputnik V	August 2020	Algeria, Argentina, Armenia, Bahrain, Belarus, Bolivia, Egypt, Gabon, Ghana, Guatemala, Guinea, Guyana, Honduras, Hungary, Iran, Kazakhstan, Kyrgyzstan, Lebanon, Mexico, Mongolia, Montenegro, Myanmar, Nicaragua, Pakistan, Palestine, Paraguay, Republika Srpska, Russia, Saint Vincent and the Grenadines, San Marino, Serbia, Syria, Tunisia, Turkmenistan, United Arab Emirates, Uzbekistan, Venezuela
Sinopharm & Wuhan Institute of Biological Products	September 2020	China, UAE
Sinopharm & Beijing Institute of Biological Products	September 2020 (EUA) December 2020 (Final approval)	Argentina, Bahrain, Cambodia, China, Egypt, Hungary, Iraq, Jordan, Laos, Macau, Morocco, Nepal, Pakistan, Peru, Senegal, Serbia, Seychelles, UAE, Zimbabwe
Vector State Virology & Biotechnology Center	October 2020	Russia, Turkmenistan
Pfizer & BioNTech	December 2020	Albania, Andorra, Argentina, Aruba, Australia, Bahrain, Canada, Chile, Colombia, Costa Rica, Ecuador, EU, Faroe Islands, Greenland, Iceland, Iraq, Israel, Japan, Jordan, Kuwait, Liechtenstein, Malaysia, Mexico, Monaco, New Zealand, North Macedonia, Norway, Oman, Panama, Philippines, Qatar, Saint Vincent and the Grenadines, Saudi Arabia, Serbia, Singapore, Switzerland, UAE, UK, US, Vatican City, WHO
mRNA-1273 (Moderna)	December 2020	Canada, EU, Faroe Islands, Greenland, Iceland, Israel, Liechtenstein, Norway, Qatar, Saint Vincent and the Grenadines, Singapore, Switzerland, United Kingdom, United States
AstraZeneca	January 2021	Argentina, Bahrain, Bangladesh, Barbados, Brazil, Chile, Dominican Republic, Ecuador, El Salvador, Egypt, EU, Guyana, Hungary, India, Iraq, Maldives, Mauritius, Mexico, Morocco, Myanmar, Nepal, Nigeria, Pakistan, Philippines, Saint Vincent and the Grenadines, South Africa, South Korea, Sri Lanka, Taiwan, Thailand, UK, Vietnam
Covaxin	January 2021	India
CoviVac	January 2021	Russia
Janssen Vaccine (Johnson & Johnson)	February 2021	Saint Vincent and the Grenadines, Bahrain, United States

Only a few COVID-19 vaccines passed phase III clinical studies. Regardless of their approval, the safety and efficacy of these vaccines in pregnant women and children remain to be elucidated. It is also of interest to know whether these vaccines are effective against various strains of SARS-COV-2. This is very crucial following the global surge in COVID-19 cases and the identification of new variants, which are thought to have rapid transmissibility and infectivity. With many countries have already started to roll out the vaccination programs, only 4 vaccines, namely Pfizer, Moderna, Oxford, and Sinopharm, are currently in phase IV clinical studies to monitor the long-term protection and side effects of these vaccines.

## Acknowledgements

We thank Professor Assam El-Osta, Central Clinical School, Monash University, Australia for his valuable comments.

## Ethical Issues

Non applicable.

## Conflict of Interest

Authors declare no conflict of interests.
